# Biomass pyrolysis-derived aqueous phase as a laccase inducer in *Pleurotus ostreatus*: laccase production, properties, and applications

**DOI:** 10.1186/s40643-026-01037-0

**Published:** 2026-03-26

**Authors:** Elmin Rahic, Cooper J. Hess, Robert C. Brown, Zhiyou Wen

**Affiliations:** 1 Bioeconomy Institute, Iowa State University, 617 Bissell Rd., Ames, IA 50011 USA; 2 Food Safety and Enteric Pathogens Research Unit, National Animal Disease Center, Agricultural Research Service, USDA, Ames, IA USA; 3 Department of Food Science and Human Nutrition, Iowa State University, 1041 Food Sciences Building, 536 Farm House Ln, Ames, IA 50011 USA

**Keywords:** Laccase, White-rot fungi, Pyrolysis, Bio-oil, Aqueous phase, Fermentation, Mediator

## Abstract

The aqueous phase (AP) generated during biomass pyrolysis is often considered a waste product due to its dilute and toxic nature, making it difficult to upgrade. This study explores the potential of using AP as a laccase inducer for the white-rot fungus, *Pleorotus ostreatus*, and as a mediator in laccase-catalyzed reactions. As an inducer, AP increased laccase production from *P. ostreatus* to 570 U/g, outperforming copper, a common inducer, by almost 180%. A maximum laccase yield of 955 U/g was achieved when *P. ostreatus* was co-induced by both AP and copper. Characterization of the AP-induced laccase revealed greater pH tolerance relative of this enzyme compared to copper-induced laccase. The AP-induced laccase was further evaluated for various applications. Laccase alone was effective in decolorizing coomassie blue dye, increasing saccharification yield from prairie biomass, and detoxifying tetracycline. When laccase was mediated with AP, the enzyme was also capable of decolorizing crystal violet dye, demonstrating additional benefit of AP to mediate laccase-based oxidation reactions with certain substrates. Overall, these findings suggest that using AP to induce laccase production, and potentially mediate the laccase-based reactions, could be a promising method to valorize this byproduct from biomass pyrolysis.

**Graphical abstract:**

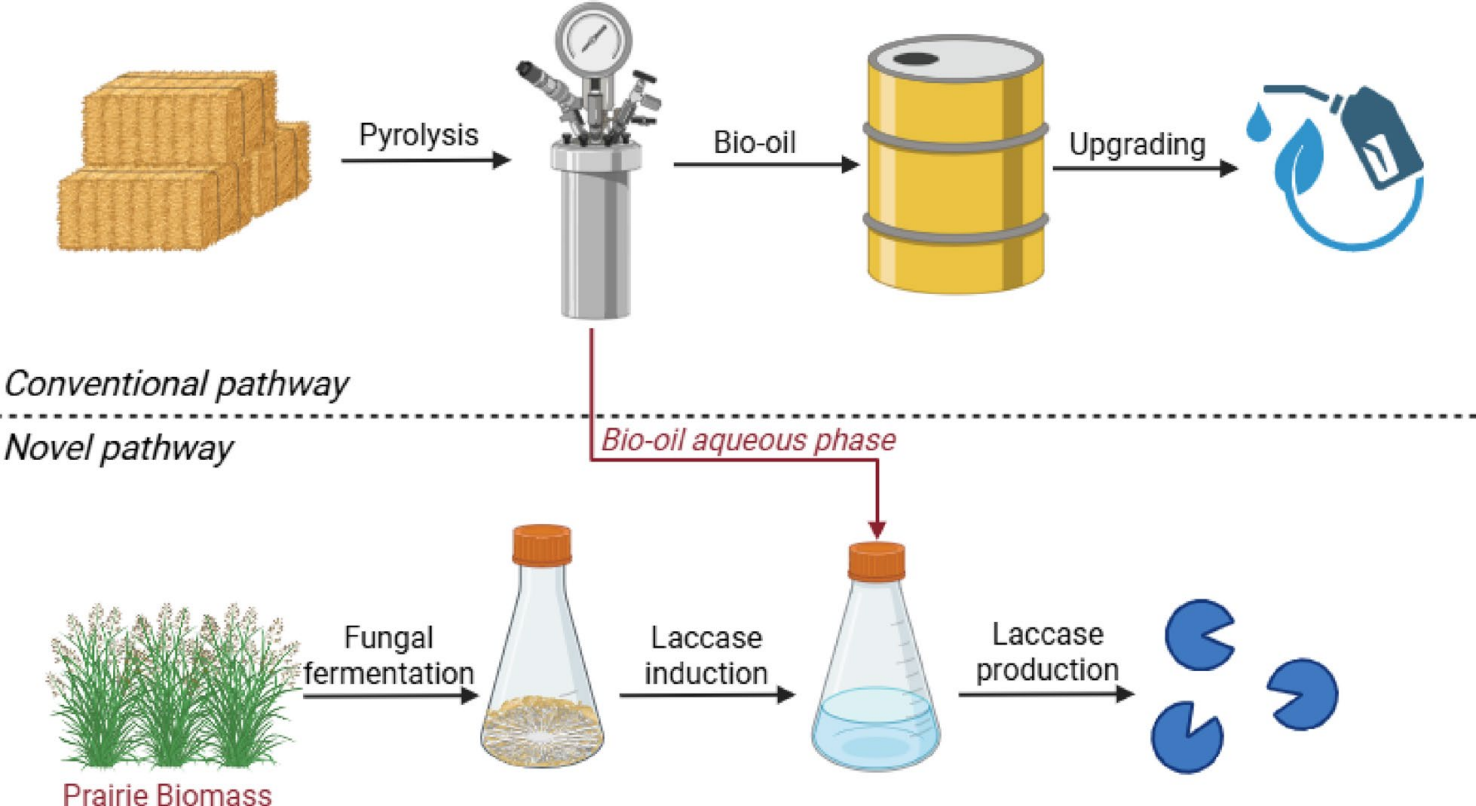

## Introduction

Pyrolysis has become an increasingly attractive technology for the conversion of lignocellulosic biomass into fuels, chemicals, and biochar. Fast pyrolysis of biomass, the process of treating biomass at high temperatures (300–700 °C) with rapid heating rates (10–200 °C/s) in an oxygen-free environment, yields three products: syngas, biochar, and a crude liquid. Syngas is a culmination of various combustible gas species, which is typically burned to provide process heating. Biochar is a carbon-rich material that has been extensively studied for its value in soil amendment, carbon sequestration, and anaerobic digestion (Jeyasubramanian et al. 2021). The primary product of fast pyrolysis is a crude liquid (up to 75 wt%) consisting of a heavy phase (bio-oil) and an aqueous phase (AP), of which the bio-oil is the more desirable product as it contains properties that make it attractive for use as a drop-in fuel (Bridgwater 2012; Laird et al. 2009; Wang et al. 2020). Despite its potential, bio-oil is a crude resource that requires substantial processing to upgrade into a drop-in fuel (Zhang et al. 2013).

Researchers at Iowa State University have developed a stage fractionation system to separate the heavy ends and the pyrolysis-derived AP from the crude liquid. The heavy ends consist of viscous sugars and lignin oligomers, while the AP is a solution containing carboxylic acids, phenols, furfural, and other organic compounds (Pollard et al. 2012). The current global market size for pyrolysis oil is estimated at $1.7 billion USD with projected rapid growth over the near future (Mordor Intelligence 2026). As one of the major products from biomass pyrolysis, there is a strong incentive to developing value-added applications for AP. Currently, AP is largely considered a liability in industrial techno-economic models, where it is commonly designated for wastewater treatment or combusted for process heat rather than being condensed for potential value-added recovery (Black et al. 2016; Ganguly et al. 2022). However, certain commercial applications for AP may exist as a form of pyroligneous acid or ‘wood vinegar’ (Grewal et al. 2018; Zheng et al. 2023). The high carboxylic acid content in AP has piqued interest in its use for heterotrophic bioprocessing applications, such as microalgae cultivation, anaerobic digestion, and yeast fermentation (Lian et al. 2012; Zhao et al. 2016; Zhou et al. 2019). The phenolic compounds present in AP are generally toxic to microorganisms even when detoxification techniques are used (Zhao et al. 2016). However the phenolics-containing AP may be a good substrate for white-rot fungi, as this group of fungi can secrete powerful oxidative enzymes, such as laccase, to degrade and catabolize lignin-derived aromatics (del Cerro et al. 2021).

Laccase, as the most prominent oxidative enzyme, is a multi-copper oxidase that catalyzes oxidation reactions on a variety of phenolic and other aromatic compounds. Laccase is currently used in the textile industry and has been studied for applications in other areas such as bioremediation, biosensors, and biomass delignification (Khatami et al. 2022). In the context of biomass delignification, laccase acts as a biological pretreatment agent by depolymerizing lignin through the oxidation of its phenolic subunits. This process increases lignin porosity and provides hydrolytic enzymes with greater accessibility to the structural carbohydrates, thereby improving saccharification yields (Rencoret et al. 2017). Reports on the current global market size for laccase vary significantly, from $3 million to $400 million USD, with varying expected growth rates (Strategic Market Research 2025; Market Reports World 2025). However, the most recent patent review by Zerva et al. (2019) has demonstrated increasing interest and investment from the commercial sector over the past decade. White-rot fungi are reported to be one of the most efficient producers of laccase (Wang et al. 2019), however, there are several barriers impeding the commercial viability of fungal laccases, including high production cost, poor thermostability, low activity at neutral or alkaline pH, and expensive redox mediators (Mani et al. 2018; Wang et al. 2021; Zerva et al. 2019). The phenolic compounds within AP may induce laccase production in white-rot fungi by acting as structural mimics of lignin subunits (Giatti Marques De Souza et al. 2004). As such, we hypothesize that AP may be a suitable laccase inducer in white-rot fungi to increase laccase production yields, potentially reducing the cost of production.

In addition to inducing laccase production, we hypothesize that the phenolics in AP could also serve as a redox mediator for laccase-catalyzed reactions. Some reactions cannot be catalyzed by laccase directly due to its low redox potential or steric hinderance. In this case, certain chemicals (redox mediators) can be added to act as an electron shuttle between laccase and the target compound. These redox mediators are oxidized by laccase, and then react with the target compound in place of the laccase. Synthetic chemicals, such as 2,2'-azino-bis(3-ethylbenzothiazoline-6-sulfonic acid) (ABTS) and 1-hydroxybenzotriazole, are powerful redox mediators but are expensive (Mani et al. 2018). Certain phenols contained in AP have been reported to mediate laccase-catalyzed reactions in isolation (Camarero et al. 2005). For example, Rico et al. (2014) investigated the pretreatment of *Eucalyptus globulus* by *Myceliophthora thermophila* laccase. They reported a 20% increase in saccharification yield from laccase alone, and when methyl syringate was added, a 41% increase in saccharification yield was obtained. However, the use of the complex AP for this purpose remains unexplored. This study is the first to explore the use of AP for enzyme production and as a mediator for laccase-catalyzed reactions. The aim of this study is to explore a novel approach to valorizing AP by evaluating its role as a laccase inducer for the white-rot fungus *Pleurotus ostreatus* and as a redox mediator for laccase-catalyzed reactions. Specifically, this study implemented response surface methodology to maximize laccase yield with AP and copper, investigated the thermal and pH tolerance of the non-induced, AP-induced, and AP + copper-induced laccases, and evaluated potential applications of AP-induced laccase in dye decolorization, biomass pretreatment, and antibiotic degradation.

## Materials and methods

### Preparation and treatment of pyrolysis-derived aqueous phase

Autothermal pyrolysis of corn stover and the crude liquid separation was performed based on the method reported previously (Pollard et al. 2012). Briefly, corn stover was pyrolyzed in a fluidized bed reactor with the crude liquid fractionated into three distinct stage fractions. The AP was prepared from stage fraction 3 and stored at − 20 °C in 1 L Nalgene HDPE bottles until use.

Prior to use, each liter of AP solution was treated by slowly adding 140 g of lime (Ca(OH)_2_) with constant stirring at 100 rpm. The treated solution was allowed to cool to room temperature for 1 h based on the protocol reported by Zhao et al. (2015). The lime-treated solution was centrifuged at 2000 g for 5 min to separate the precipitant. The supernatant was stored at 4 °C. Table 1 provides a characterization of the AP.

**Table 1:** Characteristics of the raw AP and prairie biomass^a^

Chemical compound	AP	Prairie biomass	Unit
Moisture	43.8	3.6	wt %
Total acid number	91.7	–	mg KOH/g
Total phenolics	25.9	–	g/L gallic acid equiv
pH	2.1	–	
Volatile solids	–	93%	% dry basis
Carbon	–	44.5	wt%
Nitrogen	–	0.47	wt%
Cellulose	–	33.2	% dry basis
Hemicellulose	–	26.5	% dry basis
Lignin	–	16.8	% dry basis

^a^The parameters were determined based on the methods described in Sect. "Analyses"

### Inoculum subculture

*P. ostreatus* isolated from grain spawn purchased from a local mushroom producer in Cambridge, Iowa, was used as the laccase production strain. The strain was identified based on molecular analysis of the 18S region, showing 100% identity with 94% query coverage with *P. ostreatus* Po-13 (Accession Number: FJ379284.1). The strain was grown on potato dextrose agar (PDA) at 28 °C for 7 days prior to being used as an inoculum for fermentation experiments.

### Two-stage culture of P. ostreatus for laccase production

Laccase production by *P. ostreatus* was performed in a two-stage culture system: a fungal growth phase and a laccase induction phase. The growth phase took place in 250 mL Erlenmeyer flasks under solid-state conditions with prairie biomass being used as the substrate. The biomass was passed through a hammer mill equipped with a ¼" screen. Characteristics of prairie biomass are shown in Table 1. Moisture content of the solid substrate was adjusted to 80 wt% using a liquid medium containing: 8 g/L NH_4_SO_4_, 4 g/L KH_2_PO_4_, 1 g/L MgSO_4_, 0.2 g/L CaCL_2_, and 0.2 g/L CuSO_4_. After substrate preparation, the flasks were autoclaved at 121 °C for 20 min, and then inoculated with 1 cm mycelial plugs of *P. ostreatus* from the outer ring of growth from a PDA plate. The culture was incubated at 28 °C under static conditions for 8 days before switching to the induction stage.

The induction stage of the culture was performed by submerging the solid-state growth stage culture with sterilized water to adjust the moisture content to 97 wt%. Inducer(s) was then added to boost laccase production. The flasks were incubated on a rotary shaker (150 rpm) at 25 °C. The culture was sampled periodically for laccase yield, with ~ 12-h sampling intervals for the first three days of laccase production, followed by ~ 24–48-h intervals afterward. After maximum laccase yield was reached, the crude laccase-containing broth was centrifuged at 5,000 g for 10 min. The supernatant was further filtered through a 0.22 µm membrane and stored at 4 °C until use.

Copper sulfate and AP were evaluated as laccase inducers with the *P. ostreatus* culture. When the two inducers were jointly used, their concentrations were optimized based on a central composite design (Table 2). Laccase yield (L) was correlated as a function of copper concentration (CC) and AP concentration (AP) through a second-order polynomial equation:

1 $$\begin{array}{c}L = {\upbeta }_{0} + {\upbeta }_{1}AP + {\upbeta }_{2}CC + {\upbeta }_{3}A{\mathrm{P}}^{2} + {\upbeta }_{4}C{\mathrm{C}}^{2}+ {\upbeta }_{5}AP\cdot CC\end{array}$$

where β_0_ through β_5_ are coefficients estimated by the model.

**Table 2:** Central composite design evaluating the effect of AP and copper concentrations on laccase yield

ID#	Factor		Laccase yield (U/g)	
	AP conc. (wt%)	Copper conc. (g/L)	Actual value	Predicted value
1	0.38	0.45	380	365
2	1	0.13	560	469
3	1	0.76	247	365
4	2.5	0.00	372	510
5	2.5	0.45	970	1040
6	2.5	0.45	987	1040
7	2.5	0.45	1010	1040
8	2.5	0.45	988	1040
9	2.5	0.45	1240	1010
10	2.5	0.89	584	427
11	4	0.13	523	424
12	4	0.76	302	411
13	4.62	0.45	369	366

### Dye decolorization assays

The effectiveness of the laccase for decolorization was evaluated using three dyes: crystal violet, coomassie brilliant blue R-250, and methyl orange. Experiments were performed in tubes containing crystal violet dye (0.02 mM), coomassie blue (0.01 mM), methyl orange (0.12 mM) in citrate buffer (0.1 M, pH 5), respectively. The tubes were held in an incubator at 30 °C with continuous agitation (200 rpm). Crystal violet (λ_max_ = 595 nm), Coomassie blue (λ_max_ = 465 nm), and methyl orange (λ_max_ = 465 nm) concentrations were measured calorimetrically at 0- and 12-h incubation. Decolorization efficiency was calculated according to the following formula:

2 $$\begin{array}{c}Decolorization \left(\mathrm{\%}\right)=\frac{\left({\mathrm{A}}_{0}-{\mathrm{A}}_{12}\right)}{ {\mathrm{A}}_{0} }\times 100\end{array}$$

where A_0_ and A_12_ represent absorption at 0- and 12-h incubation, respectively.

### Laccase-based treatment of prairie biomass

Treatment of prairie biomass by laccase was performed in 250 mL Erlenmeyer flasks, with experimental parameters derived from preliminary screening experiments (data not shown). Three grams of prairie biomass was added to each flask and adjusted to 80% moisture content with sterilized citrate buffer (0.1 M, pH 4.3) to mimic the pH environment of grass silage (Bernardes et al. 2019). After laccase addition, the reaction contents were mixed aseptically and incubated at the desired temperature. The flasks were incubated for 18 h, then placed in an oven to dry at 104 °C until fully dried.

Treatment efficacy was determined by measuring the saccharification yield of the prairie biomass using the NREL LAP Low Solids Enzymatic Saccharification of Lignocellulosic Biomass assay (Resch et al. 2015) with slight modifications. In short, the dried biomass was added to 0.1 M citrate buffer (pH 4.8) at 2% (w/v) loading. Cellulase enzymes from *Trichoderma reesei* ATCC 26921 (Sigma-Aldrich) were added at 30 FPU/g biomass. The mixture was held in an incubator (New Brunswick Scientific) at 50 °C and 200 rpm for 5 days. After incubation, samples were centrifuged at 10,000 g for 3 min, filtered through a 0.22 µm PES membrane, and stored at − 20 °C prior to glucose analysis.

The previously mentioned preliminary screening experiments revealed moisture content, laccase concentration, and treatment temperature as significant factors for the laccase-based treatment of prairie biomass. For this study, a central composite design was used to optimize laccase concentration and temperature. Moisture content was maintained at the optimal value (80%) determined during our preliminary screening. Each factor was evaluated at four levels (Table 3). Saccharification yield (SY) was correlated with laccase concentration (LC) and temperature (T) through a second-order polynomial equation:

3 $$\begin{array}{c}SY = {\upbeta }_{0} + {\upbeta }_{1}T + {\upbeta }_{2}LC + {\upbeta }_{3}{\mathrm{T}}^{2} + {\upbeta }_{4}L{\mathrm{C}}^{2} + {\upbeta }_{5}T\cdot LC\end{array}$$

where β_0_ through β_5_ are coefficients estimated by the model.

**Table 3:** Central composite design to model the effect of temperature and laccase concentration on the saccharification yield of prairie biomass^a^

ID#	Factor		Saccharification yield		
	Temperature (°C)	Laccase conc. (U/g)	Actual value (wt% of total glucose)	Actual improvement (%)	Predicted improvement (%)
1	25	8	10.6	12.6	8.3
2	25	44	11.8	25.6	26.6
3	25	80	11.9	26.3	26.5
4	37.5	8	11.0	17.3	14.6
5	37.5	44	13.1	38.8	34.7
6	37.5	44	12.4	31.2	34.7
7	37.5	44	12.5	32.5	34.7
8	37.5	44	12.7	34.3	34.7
9	37.5	80	12.8	35.7	36.3
10	50	8	11.0	16.6	14.2
11	50	44	12.9	36.4	36.0
12	50	80	13.5	43.3	39.3
13	60	8	10.6	11.7	9.1
14	60	44	12.3	30.5	32.2
15	60	80	12.7	35.0	36.9
16	25	0	9.4	-0.1	1.8
17	37.5	0	9.8	4.1	7.7
18	50	0	9.8	4.1	6.9
19	60	0	9.5	1.0	1.4

^a^ Data are presented as means of two replicates

### Laccase treatment of tetracycline

Tetracycline (100 mg/L) was incubated with laccase (45 U/mL) in the presence or absence of AP (0, 5, 10, or 15 µL/mL) at pH 6 and 30 °C for 24 h. Controls (laccase-free) were incubated side-by-side. To study the efficacy of laccase treatment on tetracycline detoxification, growth inhibition tests were performed to evaluate the antimicrobial activity of the treated and untreated tetracyclines at 10 mg/L using *Pseudomonas putida* KT2440 as the model strain. Growth inhibition tests were conducted in 24-well plates containing Luria–Bertani medium and incubated at 37 °C without agitation. Cell growth was monitored by measuring absorbance of the cell growth medium at 600 nm with a GENESYS 180 UV–Vis Spectrophotometer (Thermo Scientific) over the course of 24 h. All conditions were evaluated in triplicate. The efficacy of laccase treatment was evaluated by comparing the 24-h growth of *P. putida* against a control that was not exposed to tetracycline, based on the following formula:

4 $$\begin{array}{c}Microbial growth \left(\mathrm{\%}\right)=\frac{\left({\mathrm{X}}_{24}-{\mathrm{X}}_{0}\right) }{ \left({\mathrm{C}}_{24}-{\mathrm{C}}_{0}\right) }\times 100\end{array}$$

where X_0_ and X_24_ represent growth of the treatment groups at 0- and 24-h incubation, respectively, and C_0_ and C_24_ represent growth of the controls without tetracycline at 0- and 24-h, respectively.

### Analyses

Laccase activity was measured calorimetrically through the oxidation of 0.2 mM ABTS (ε = 36,000 M^−1^ cm^−1^) at 420 nm in citrate–phosphate buffer (0.1 M, pH 3) at 25 °C, and calculated using the suggested equation by Baltierra-Trejo et al. (2015). One unit of laccase activity was defined as the quantity of laccase required to oxidize 1 µmol of ABTS per min. Laccase activity for each sample was taken as the average of duplicates and reported as units per gram of prairie biomass on a dry matter basis. The phenolic content of the fermentation broth during laccase production was measured via the Folin Ciocalteu method with gallic acid as the standard. During saccharification experiments, the glucose extracted from the prairie biomass was measured using a Thermo Fisher Scientific/Dionex Ultimate 3000 HPLC. Information about this method can be found in (Johnston & Brown 2014).

Lignin, cellulose, and hemicellulose content were determined by Celignis Biomass Analysis Laboratory (Limerick, Ireland) using the NREL Laboratory Analytical Procedure (LAP) TP-510–42618 (Sluiter et al. 2012). Carbon and nitrogen analysis was performed using an Elementar vario MICRO cube. All statistical analyses in this study were performed using JMP16.

## Results and discussion

### Inducing laccase production with AP

**Fig. 1:**
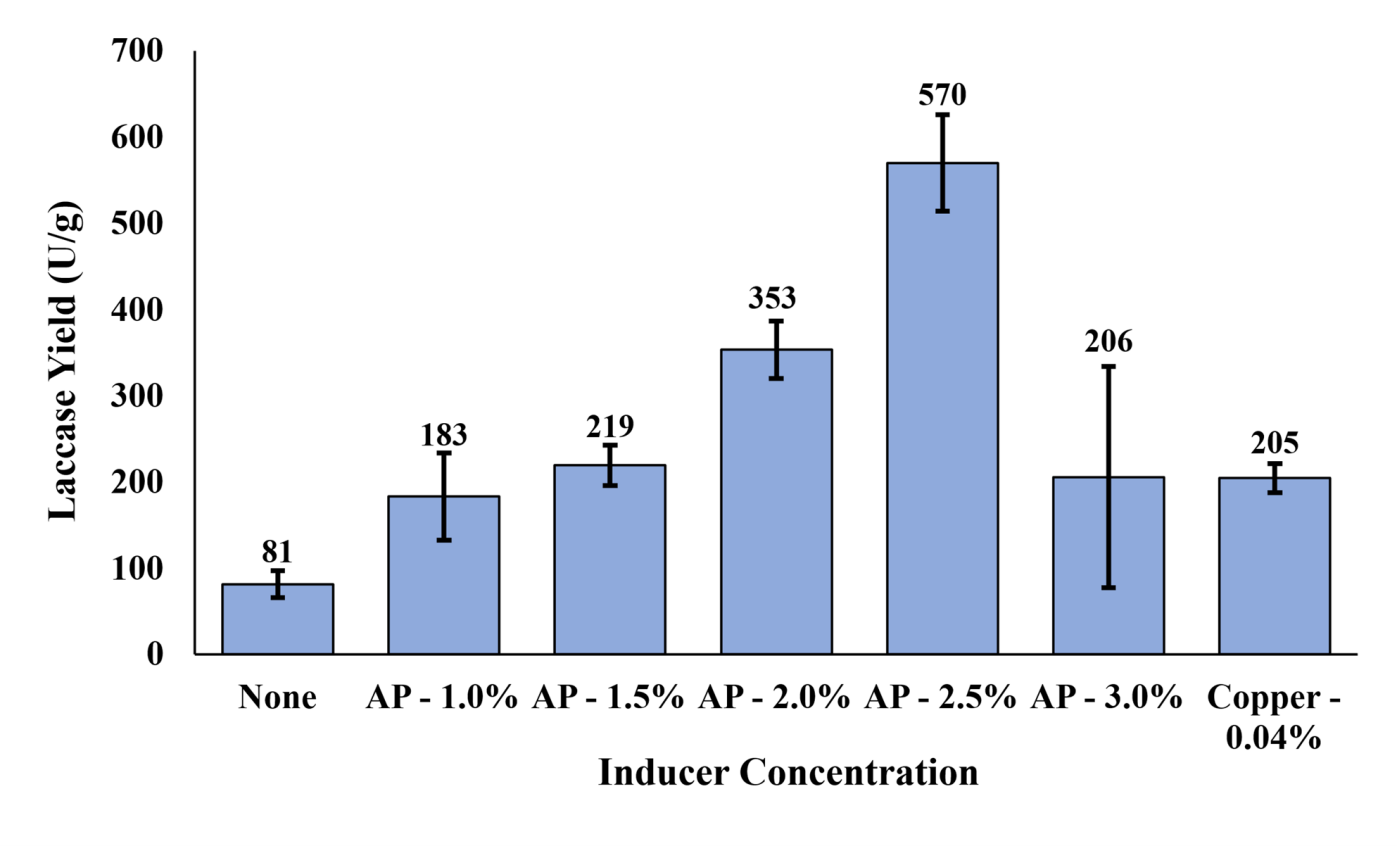
Laccase yield by *P. ostreatus* culture induced by AP at different concentrations and copper at its optimal concentration of 0.04%. Results are presented as the mean of three replicates and error bars represent standard deviations

AP was evaluated as an inducer for laccase production by *P. ostreatus*. As shown in Fig. 1, compared to the AP-free culture, AP addition increased the laccase yield for all concentrations evaluated. Laccase yield progressively increased with AP from 1.0 to 2.5% (v/v) and peaked at 570.0 U/g. When AP increased to 3.0%, laccase production dropped significantly with greater variability. This sharp decline suggests that 3.0% AP addition represents a threshold where the toxic effects of AP begin to inhibit fungal activity. The high variability may point to a ‘tipping point’ effect where, at this concentration of AP, minor differences across replicates can lead to inconsistent yields and adaptation to inhibitors. While the toxicity of AP to filamentous fungi has not yet been explored, research on AP and compounds commonly contained within AP have reported several mechanisms behind AP inhibition, including: cell membrane disruption from hydrophobic (typically phenolic) compounds (Jin et al. 2017), damage to DNA, proteins, and cell organelles from furans through an overaccumulation of reactive oxygen species (Wierckx et al. 2011; Lian et al. 2010), and environmental and intracellular acidification from carboxylic acids (Jarboe et al. 2013; Liang et al. 2013). Although the overliming treatment employed in this study (Sect. "Preparation and treatment of pyrolysis-derived aqueous phase") likely mitigated the acidification caused by the carboxylic acids, this treatment does not fully remove the toxicity of the other aforementioned compounds (Zhao et al. 2015). Figure 1 also shows the laccase yield when induced by copper sulfate at its optimal concentration of 0.04% (determined in our preliminary studies, data not shown). Laccase yields at 1.0, 1.5, and 3.0% AP were comparable to copper, while 2.0% and 2.5% AP yielded 73% and 179% more laccase than copper, respectively. Laccase production times differed based on inducer type and concentration, with copper induction reaching maximum laccase production within 72 h, and the AP induction requiring 84–300 h, with higher AP concentrations requiring more time. These results suggest that AP is a strong inducer for laccase production. While a molecular analysis into determining the mechanism behind AP induction was beyond the scope of this study, earlier work suggests that the induction of laccase by AP may be explained as a stress response from *P. ostreatus*. Certain *P. ostreatus* strains have been found to contain a number of unique laccase promoters with a diverse array of response elements, including xenobiotic response elements, which respond to phenolic or aromatic compounds to start transcription. Notably, xenobiotic response elements were found in both of the most active laccase promoters (POXC and POXA3) (Piscitelli et al. 2011). It should be noted that fungal biomass results were not reported in this work due to the challenges of direct separation of fungal biomass from the solid substrate. However, we recognize the importance of this data and the existence of some indirect methods based on metabolic indicators to estimate fungal growth. This warrants future studies evaluating the fungal growth as a response to AP.

AP and copper were further evaluated as co-inducers to maximize laccase production. AP and copper concentrations were optimized for laccase yield through a central composite design. Table 2 lists the design matrix with the actual laccase yield for each run. Table 4 lists the parameter estimates for Eq. (1) and their significance based on F-test statistics and their corresponding *p*-values. In this study, model terms were considered statistically significant if their *p*-value was less than 0.05.

**Table 4:** Parameter estimates and F-test statistics for modeling laccase yield (Eq. 1) ^a^

Coefficient	Variable	Estimate	F-value	*P*-value
β_0_	Constant	-371		
β_i_	AP Conc	727	< 0.01	0.996
β_j_	Copper Conc	2352	0.47	0.516
β_ii_	AP Conc.^2^	-150	39.5	0.0004
β_jj_	Copper Conc.^2^	-2875	28.4	0.001
β_ij_	AP Conc. × Copper Conc	48.4	0.10	0.755

^a^ *P*-values correspond to the F-test statistic. Model parameters were considered statistically significant if their *p*-value was below 0.05. Units for laccase yield are reported in U/g of prairie biomass

**Fig. 2:**
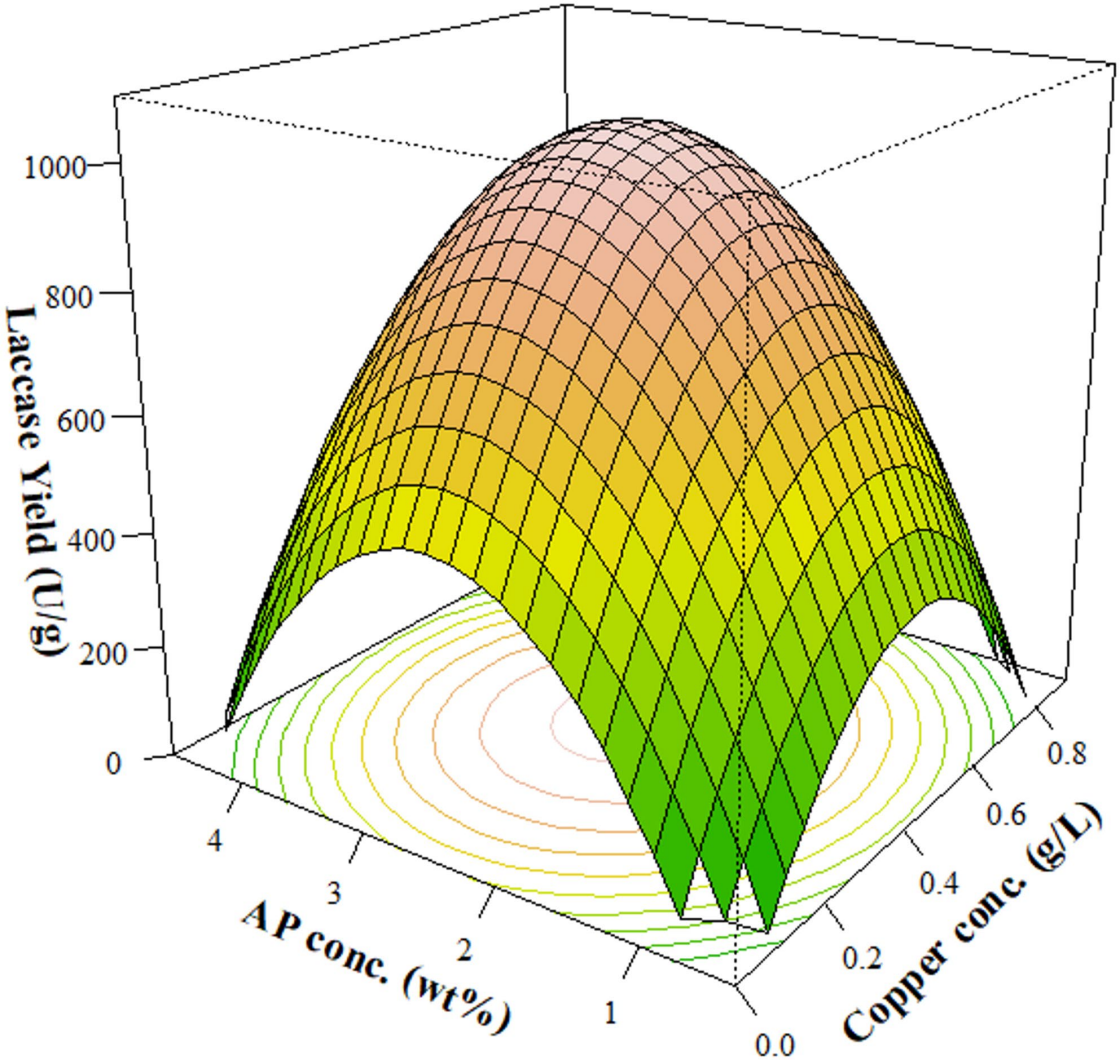
Surface response of laccase yield from *P. ostreatus* as a function of AP and copper concentrations

The quadratic terms (Cu^2^ and AP^2^) showed statistical significance, while the linear terms (Cu and AP) and their interaction (AP × Cu) were deemed insignificant (Table 4). The model R^2^ (0.90) implies a moderate level of variance from the experimental data; however, both the model significance (*p* = 0.002) and insignificant lack-of-fit test (*p* > 0.05) suggest an adequate model fit and significance. Figure 2 depicts the surface response of the laccase yield. Based on Eq. (1) and Fig. 2, a maximum laccase yield of 1040 U/g was predicted at 2.5% AP and 0.043% copper. This is substantially higher than the laccase yields induced from the individual inducers (Fig. 1), signifying the benefit of using co-inducers for laccase production.

**Fig. 3:**
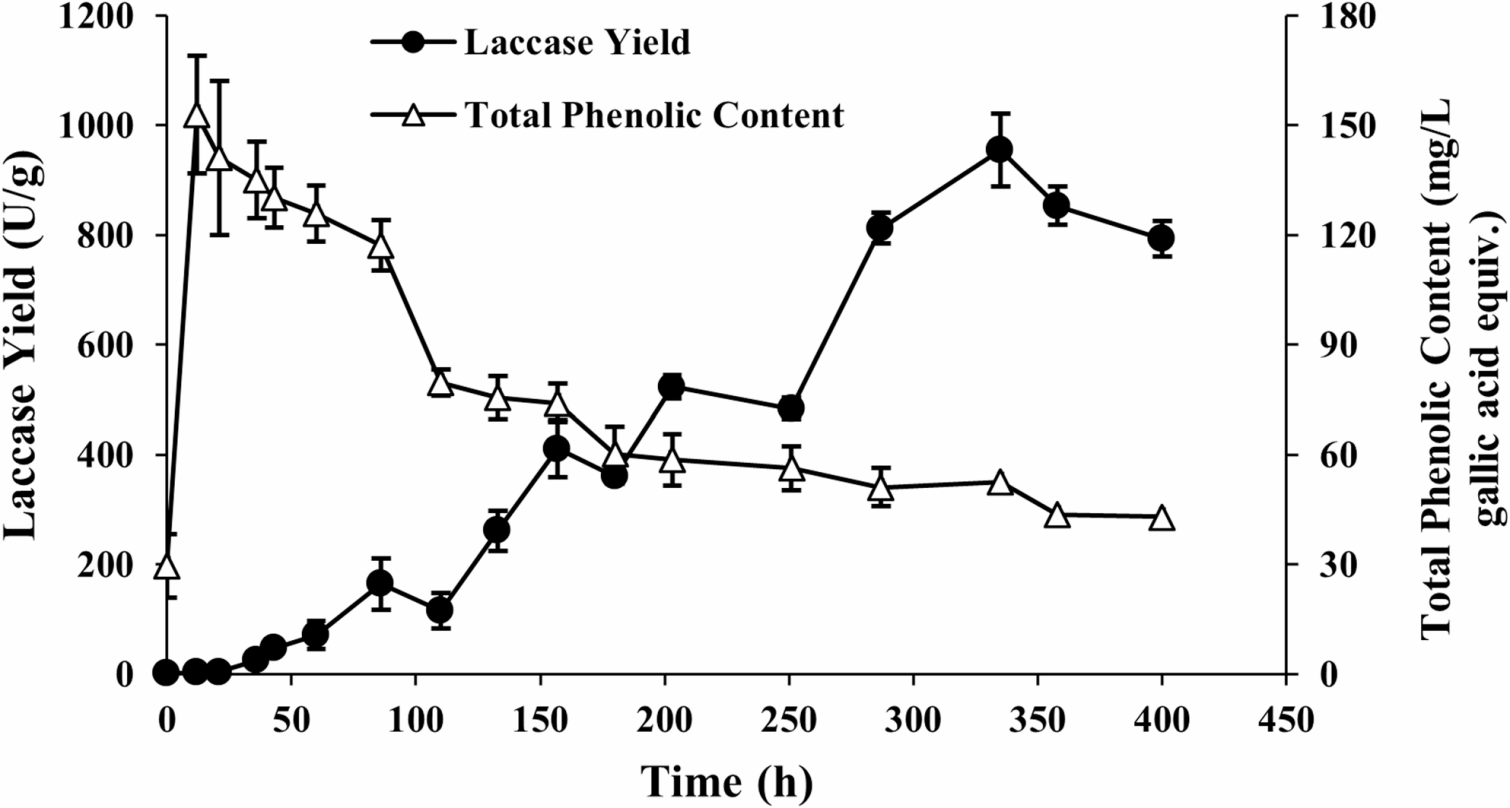
Comparison of laccase yield and total phenolic content during the induction phase under optimized conditions. The sample at time zero was taken immediately prior to the addition of the pyrolysis-derived aqueous phase (AP) in order to evaluate the total phenolic load provided by the AP. Results are presented as the mean of three replicates, with error bars representing standard deviations

Laccase production was further evaluated by culturing *P. ostreatus* under the predicted optimal AP and copper concentrations. Figure 3 illustrates the time-course laccase yield and total phenolic content (TPC) in the culture under the optimized conditions. The maximum laccase yield (955 U/g at 335-h) was 8.1% different from (less than) the predicted yield, indicating that the prediction by Eq. (1) is reliable. The TPC showed an inverse relationship from the laccase yield, peaking immediately after AP addition and progressively decreasing over time. A 72% reduction in TPC was observed over the course of laccase production, reducing the TPC to concentrations comparable to those prior to AP addition. Tsioulpas et al. (2002) reported similar findings a significant removal of phenolic compounds during laccase production. Interestingly, the depletion of phenolics diminished substantially after 180 h. We hypothesize that this plateau is the result of oxidative coupling of monomeric phenols by laccase into larger oligomers, which are more recalcitrant to further degradation (Strong & Claus 2011). These findings may suggest that AP is detoxified during the *P. ostreatus* culture, potentially allowing the AP (and likely the whole fermentate) to be used for further valorization via anaerobic digestion or other bioprocessing means. However, this is contingent on the availability of other AP-derived carbon sources, such as organic acids, after laccase production.

It should be noted that while the AP and copper co-induction remarkably improved laccase, the final culture pH was 8.3, which is well above the reported optimal pH for *P. ostreatus* (El-Batal et al. 2015; Prasad et al. 2005)*.* This is due to the higher pH of AP after over-liming treatment. Further exploration and optimization of fermentation pH for *P. ostreatus* culture are needed.

### Characterization of AP-induced laccase

**Fig. 4:**
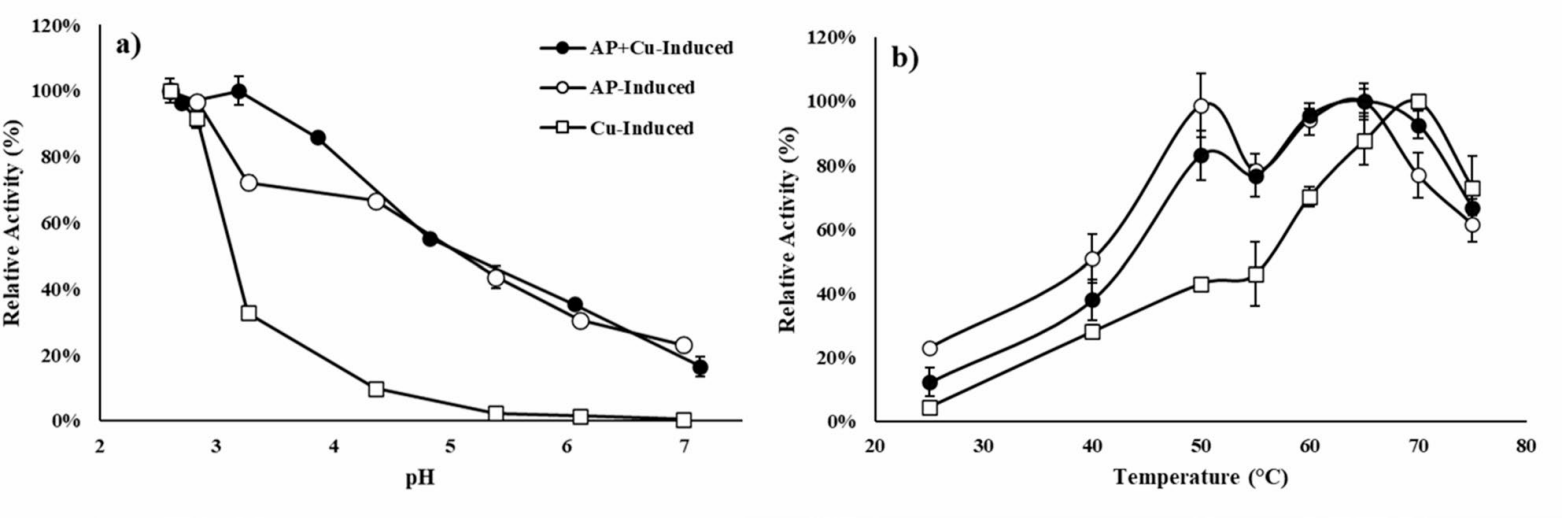
Relative activity of AP-, copper-, and AP + Cu-induced laccase at different pH (**a**) and temperature (**b**) conditions. Each laccase was produced under the optimized conditions outlined in Sect. "Inducing laccase production with AP". Laccase activities under different pH were studied at 25 °C. Laccase activity under different temperatures were studied at pH 3. Data are presented as means of three replicates. Error bars represent standard deviations

*P. ostreatus* contains laccase-encoding genes responsible for producing various laccase isozymes (Karp et al. 2012). These isozymes may exhibit different properties, thus, the laccase characteristics produced under the different induction conditions were evaluated. Figure 4 depicts the relative activity of three types of laccases, copper-induced laccase, AP-induced laccase, and AP + Cu-induced, at different pH and temperature conditions. As shown in Fig. 4a, all laccases were active at an acidic pH, which is typical of fungal laccase (Guan et al. 2018). However, both AP-induced laccases showed greater tolerance to higher pH compared to the copper-induced laccase which could be advantageous for industrial applications of this enzyme (Guan et al. 2018). In addition to the possibility of different laccase isozymes being secreted, the enhanced tolerance to alkaline conditions may be a result of post-translational modifications stemming from environmental stressors placed on the fungus (i.e. from AP). For example, higher glycosylation levels have been reported to increase active site rigidity in enzymes (Ramakrishnan et al. 2023) and protect laccase against alkaline denaturation by maintaining its structural integrity (Christensen & Kepp 2013).

Thermal properties of laccases are also of interest from industrial and environmental application perspectives (Liu et al. 2020; Mateljak & Alcalde 2021). As shown in Fig. 4b, the copper-induced laccase demonstrated the highest activity at 70 °C. The AP-induced laccase appears to reach two temperature optima, around 50 and 65 °C, potentially indicating the presence of multiple laccase isozymes. The AP + Cu-induced laccase appears to show characteristics of the other two laccases, displaying an optimum temperature at 65 °C.

The properties of the AP-induced laccases, particularly their enhanced tolerance to a broader pH range, can be advantageous for industrial applications, particularly when operational conditions fluctuate or demands differ. For example, wastewater effluents from textile and pharmaceutical sectors can vary in pH and temperature, therefore an enzyme more tolerant to environmental differences would allow for more consistent enzymatic activity and bioremediation efficacy. This trait also allows AP-induced laccases to be considered for a broader range of applications.

### Evaluating different applications for laccase

Previous results clearly show the beneficial effects of AP to induce laccase production (Fig. 1) and the desirable properties of AP- or AP + Cu-induced laccase compared to Cu-induced laccase (Fig. 4). In this work, the AP + Cu-induced laccase was studied in three application scenarios: dye decolorization, biomass pretreatment, and antibiotic degradation. In addition, AP was also evaluated as a mediator to shuttle electron between the laccase and substrates, due to the potential benefits of improving reaction rates and lowering enzyme dosage (Morozova et al. 2007).

#### Dye decolorization

In this study, laccase was tested for its efficacy in decolorizing three dyes: crystal violet, coomassie brilliant blue R-250, and methyl orange. Crystal violet and coomassie blue are triarylmethane dyes which have been widely used in the textile, cosmetic, leather, and other industries (Nakagawa et al. 2016). Several reports have also studied the decolorization of crystal violet and coomassie blue by laccase (Morales-Álvarez et al. 2018; Yang et al. 2016). Methyl orange is an azo dye, which represent up to 70% of dyestuffs used in industry (Pramanik and Chaudhuri 2018). Azo dyes are designed to be recalcitrant to aerobic degradation, which makes it difficult to biodegrade. However, some studies have reported degradation of methyl orange using laccase (Telke et al. 2010).

As shown in Table 5, this laccase alone was effective in decolorizing coomassie blue, achieving 56% decolorization with 40 U/L of laccase, but was not effective on crystal violet or methyl orange. The incorporation of AP as a mediator showed mixed effects. With coomassie blue as the substrate, AP was beneficial as a mediator at a laccase concentration of 4 U/L, but worsened decolorization at higher laccase concentrations. With crystal violet, AP as a mediator significantly improved decolorization, for a maximum decolorization of 49.8% at laccase and AP concentrations of 22 U/L and 7.5 µL/mL, respectively. Increasing AP or laccase concentration beyond this provided no benefit to decolorization. Lastly, with methyl orange, no significant decolorization was observed under any condition. With this dye, only AP (without laccase) was consistently beneficial, showing greater decolorization with increasing AP concentrations. However, this effect was marginal and only statistically significant at an AP concentration of 15 µL/mL (*p* < 0.05).

**Table 5:** Decolorization of dyes by AP + Cu-induced laccase, with and without AP as a mediator ^a^

Substrate	Laccase conc. (U/L)	AP conc. (µL/mL)		
		0	7.5	15
Coomassie blue	0	5.2 ± 1.1	18.8 ± 5.8	0.5 ± 6.8
	4	12.5 ± 7.2	29.4 ± 15.5	32.7 ± 4.5
	22	46.3 ± 2.7	37.3 ± 5.7	25.6 ± 0.9
	40	55.8 ± 3.9	28.0 ± 8.9	39.3 ± 3.0
Crystal violet	0	-2.3 ± 0.9	3.0 ± 1.6	0.7 ± 2.5
	4	-6.5 ± 2.5	21.5 ± 2.5	25.5 ± 1.5
	22	1.5 ± 3.5	49.8 ± 2.6	41 ± 4.0
	40	2.5 ± 6.3	49.0 ± 1.0	35.5 ± 4.5
Methyl orange	0	3.0 ± 1.9	4.2 ± 3.1	8.8 ± 3.6
	4	1.3 ± 3.3	-7.9 ± 4.1	0.3 ± 2.4
	22	4.6 ± 8.2	-9.9 ± 10.6	-4.6 ± 23.4
	40	-0.3 ± 1.1	-5.9 ± 6.0	-6.1 ± 11.5

^a^ Data are presented as percentages, as calculated by Eq. (2), plus or minus standard deviations

While these results demonstrate potential opportunities for this laccase in dye decolorization, practical scale-up may be limited by factors not considered in this in-vitro study. In commercial environments, laccase may be used for decolorization in wastewater treatment settings or for direct decolorization from fabrics, denim, or other solid materials. Future work should encompass these real-world environmental factors into the experimental design, as well as considering other important aspects such as the recoverability of the laccase and the necessity of redox mediators.

#### Laccase-assisted prairie biomass treatment to enhance saccharification yields

Biomass pretreatment is an area of ongoing interest for lignocellulosic-based biofuel processes. The role of fungal laccase in nature is to assist in the depolymerization of lignin, which improves the accessibility of cellulosic sugars in the plant. Laccase-based treatment of biomass has been reported with varying levels of efficacy based on laccase source, environmental conditions, substrate properties, and use of mediators (Chen et al. 2012; Heap et al. 2014). To evaluate the potential of this laccase for biomass treatment, response surface methodology (Table 3) was used to reveal the effects of temperature and laccase concentration in biomass pretreatment on the glucose yield in the subsequent saccharification of pretreated biomass, with glucose yield being a measure of the susceptibility of biomass to saccharification. Prairie biomass was chosen as a substrate for this study given the beneficial soil health, water quality, and biodiversity benefits that prairie systems provide (Dutter et al. 2025; Schulte et al. 2017), as well as its growing interest as a bioenergy crop in recent years (Olafasakin et al. 2024; Rahic et al. 2024; Wild et al. 2025).

Table 6 lists the parameter estimates for Eq. 3 and corresponding F-test statistics. All model parameters were deemed significant except the interaction between laccase concentration and temperature (*p* = 0.055), which was only slightly higher than the significance level of 0.05. The model (Eq. 3) predicts a 40.3% improvement in saccharification yield under optimal conditions of a laccase concentration of 68.1 U/g and a temperature of 48.3 °C. The model statistics suggest good fit of the experimental data, with an R^2^ of 0.97, a low root mean squared error of 3.04%, and an insignificant lack of fit test (*p* > 0.05).

**Table 6:** Parameter estimates and F-test statistics for modeling the improvement in prairie biomass saccharification yield

Coefficient	Variable	Estimate	F-value	*P*-value
β_0_	Constant	-30		
β_i_	Temperature	1.8	5.5	0.036
β_j_	Laccase	0.78	320	< 0.0001
β_ii_	Temperature^2^	-0.021	16	0.0015
β_jj_	Laccase^2^	-0.0071	48	< 0.0001
β_ij_	Temperature laccase	0.0038	4.4	0.055

**Fig. 5:**
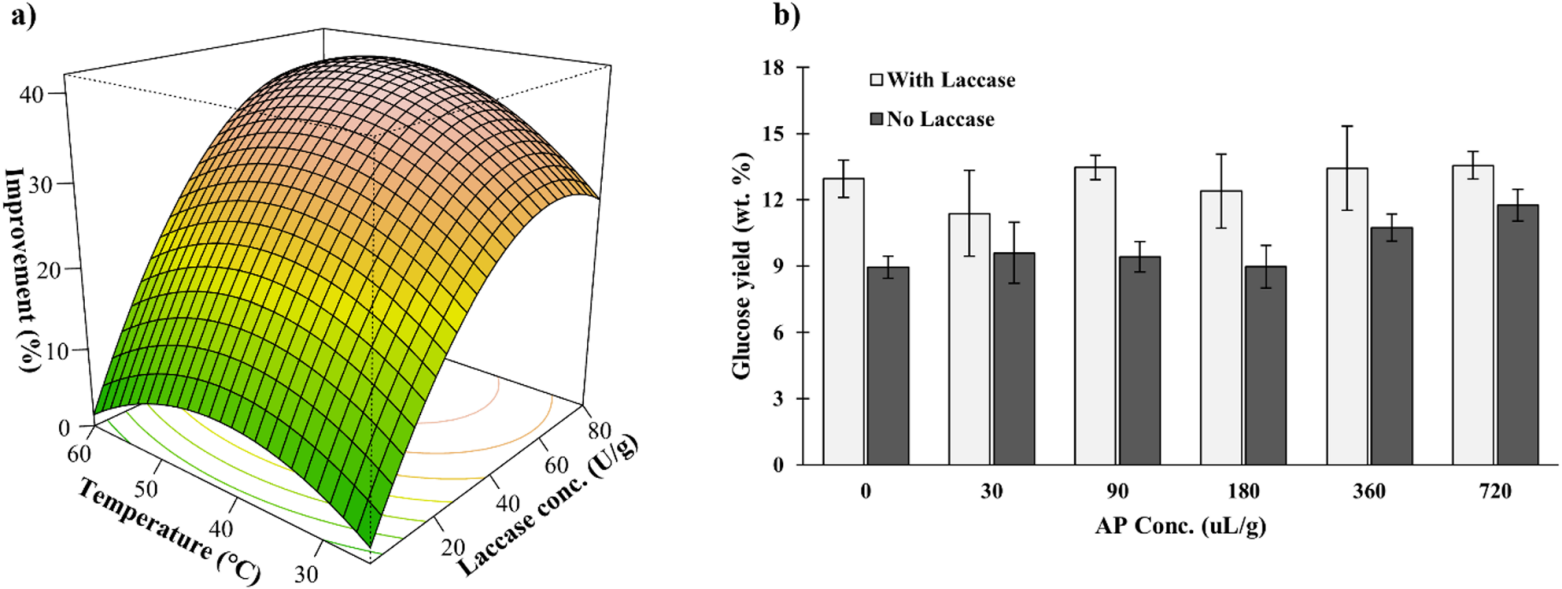
**a** Three-dimensional surface plot showing the improvement in prairie biomass saccharification yield relative to untreated biomass as a function of AP + Cu-induced laccase concentration and temperature. **b** Effect of AP concentration in biomass pretreatment at the optimal temperature of 48.3 °C in presence or absence of laccase on glucose yield in the subsequent saccharification of pretreated biomass. Data are presented as means of three replicates with error bars representing standard deviations

Figure 5a depicts the response surface plot of prairie saccharification yield improvement as response and laccase concentration and temperature as variables. Laccase concentration had a significant effect on saccharification yield, showing substantial improvements even at low temperatures. The laccase-based treatment was repeated under the optimal conditions as predicted by the model (Fig. 5b). It shows a saccharification yield of 13.0 wt% was achieved under the optimized condition, resulting in a 41% increase in yield relative to untreated prairie biomass (9.2 wt%). This is within 2.7% of the predicted improvement, indicating a high level of reliability with this model. AP was also investigated as a mediator under different concentrations (Fig. 5b). For all AP concentrations evaluated, biomass treated with laccase showed greater saccharification yields relative to those that did not receive laccase, but this effect was not statistically significant at each AP concentration. Additionally, AP addition showed no benefit as a mediator for laccase (*p* > 0.05). However, in absence of laccase, the correlation between glucose yield and AP concentration is statistically significant (p < 0.05); improvements of 20% and 31.4% in saccharification yield were observed for AP concentrations of 360 and 720 µL/g, respectively. While the saccharification yields confirm the efficacy of laccase treatment, further characterization of the prairie biomass (e.g. using FTIR or SEM) could be beneficial to visualize and verify specific functional group modifications on the lignin.

These findings highlight the potential for the AP-induced laccase to improve the efficacy of enzymatic saccharification for biofuel production. Commercial cellulase and other saccharifying enzyme cocktails do little to alter the structure of lignin and can suffer from unproductive binding onto lignin, preventing the enzyme from reacting with cellulose (Siqueira et al. 2017). As laccase can facilitate the modification and degradation of lignin, its inclusion can help reduce enzyme loss and consequently improve saccharification rates and yields. However, a detailed economic assessment is needed to confirm whether laccase addition is cost-competitive at scale.

#### Antibiotic detoxification

Antibiotics and other pharmaceutical contaminants pose a severe threat to ecological systems and human health. Antimicrobial resistance has been declared a global public health crisis and has been responsible for the death of millions worldwide (World Health Organization 2014). Antibiotic detoxification could result in the reduction of antimicrobial resistance occurrences, potentially saving many lives. In this work, tetracycline was used as model antibiotic to evaluate the efficacy of this laccase on antibiotic detoxification.

**Fig. 6:**
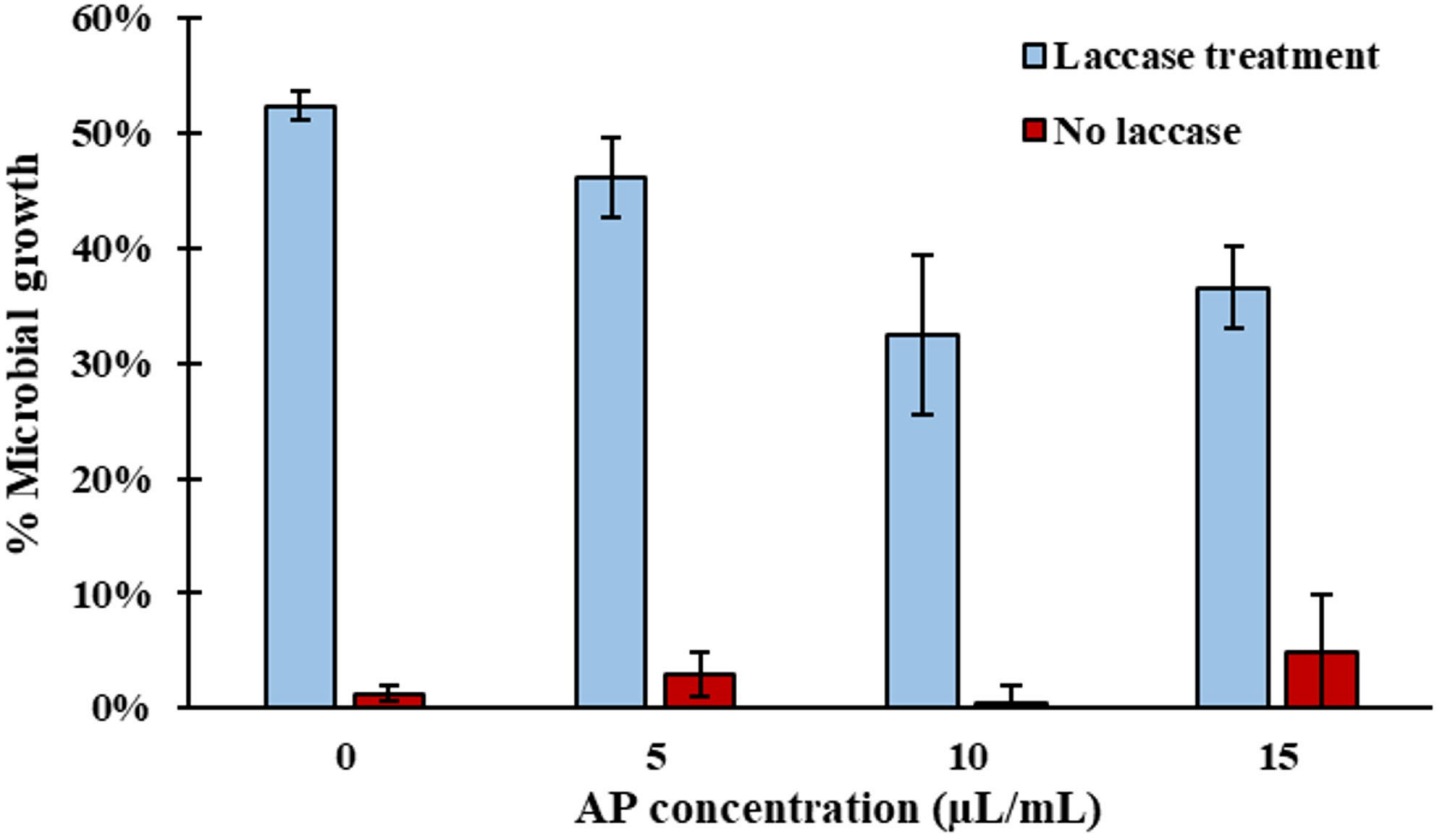
Microbial growth of *P. putida* at different AP concentrations, as calculated by Eq. (4). Data are presented as means of three replicates with error bars representing standard deviations

The detoxification of tetracycline was evaluated using growth inhibition assays with *P. putida*. As shown in Fig. 6, laccase treatment alone significantly increased growth of *P. putida* relative to untreated tetracycline*,* indicating that laccase was successful in suppressing the antimicrobial properties of tetracycline. Other researchers have also reported laccase from different sources to be effective in tetracycline removal (Harguindeguy et al. 2024; X. Wang et al. 2023; Yang et al. 2017). Tetracycline treated with both laccase and AP as a mediator also showed lower growth suppression relative to the untreated tetracycline. However, the AP did not benefit laccase performance, nor was it beneficial without laccase. In fact, incorporating AP as a mediator worsened *P. putida* growth at 10 and 15 µL/mL (*p* < 0.05).

These results show significant tetracycline-degradation potential for the AP-induced laccase. Due to the nature of laccase’s low substrate specificity, it is possible that the AP-induced laccase would also be effective in the degradation of other antibiotics and pharmaceutical compounds, as they often contain phenolic or aromatic functional groups. However, antibiotics typically enter the environment through various wastewaters and livestock manure, which often exist in anaerobic environments due to their high biological oxygen demand. As laccase requires oxygen as an electron acceptor, its activity will be restricted in these anaerobic conditions. Overcoming this challenge will be critical for future development in laccase-based bioremediation.

## Conclusion

This study has demonstrated a promising route towards the valorization of AP via the production of laccase from *P. ostreatus*. A maximum laccase yield of 955 U/g was achieved when coupling AP and copper as laccase inducers. The AP-induced laccases exhibited greater pH tolerance relative to the copper-induced laccase. This laccase was also found to be effective in the bioremediation of tetracycline, improving saccharification yields in prairie biomass, and in the decolorization of certain dyes. Lastly, AP was found to be an effective laccase mediator in the decolorization of crystal violet and coomassie blue dye, but showed no benefit for other applications in this study. Future work should investigate the mechanistic role that AP plays in inducing laccase from *P. ostreatus*, as well as further optimization of laccase production.

## Data Availability

Data will be made available upon request.

## Abbreviations

AP: Pyrolysis-derived aqueous phase
ABTS: 2,2'-Azino-bis(3-ethylbenzothiazoline-6-sulfonic acid)
PDA: Potato dextrose agar
L: Laccase yield
CC: Copper concentration
SY: Saccharification yield
LC: Laccase concentration
T: Temperature
TPC: Total phenolic content
